# Referrals to a regional allergy clinic - an eleven year audit

**DOI:** 10.1186/1471-2458-10-790

**Published:** 2010-12-29

**Authors:** Ray B Jones, Paul Hewson, Edward R Kaminski

**Affiliations:** 1 Faculty of Health, University of Plymouth, 3 Portland Villas, Plymouth PL4 8AA, UK; 2 Department of Statistics, University of Plymouth, Kirkby Place, Plymouth PL4 8AA, UK; 3 Department of Clinical Immunology & Allergy, Derriford Hospital, Plymouth PL6 8DH, UK

## Abstract

**Background:**

Allergy is a serious and apparently increasing public health problem yet relatively little is known about the types of allergy seen in routine tertiary practice, including their spatial distribution, co-occurrence or referral patterns. This study reviewed referrals over an eleven year period to a regional allergy clinic that had a well defined geographical boundary. For those patients confirmed as having an allergy we explored: (i) differences over time and by demographics, (ii) types of allergy, (iii) co-occurrence, and (iv) spatial distributions.

**Methods:**

Data were extracted from consultant letters to GPs, from September 1998 to September 2009, for patients confirmed as having an allergy. Other data included referral statistics and population data by postcode. Simple descriptive analysis was used to describe types of allergy. We calculated 11 year standardised morbidity ratios for postcode districts and checked for spatial clustering. We present maps showing 11 year rates by postcode, and 'difference' maps which try to separate referral effect from possible environmental effect.

**Results:**

Of 5778 referrals, 961 patients were diagnosed with an allergy. These were referred by a total of 672 different GPs. There were marked differences in referral patterns between GP practices and also individual GPs. The mean age of patients was 35 and there were considerably more females (65%) than males. Airborne allergies were the most frequent (623), and there were very high rates of co-occurrence of pollen, house dust mite, and animal hair allergies. Less than half (410) patients had a food allergy, with nuts, fruit, and seafood being the most common allergens. Fifteen percent (142) had both a food and a non-food allergy. Certain food allergies were more likely to co-occur, for example, patients allergic to dairy products were more likely to be allergic to egg.

There were age differences by types of allergy; people referred with food allergies were on average 5 years younger than those with other allergies, and those allergic to nuts were much younger (26 Vs 38) than those with other food allergies.

There was clear evidence for spatial clustering with marked clustering around the referral hospital. However, the geographical distribution varied between allergies; airborne (particularly pollen allergies) clustered in North Dartmoor and Exmoor, food allergies (particularly nut allergies) in the South Hams, and on small numbers, some indication of seafood allergy in the far south west of Cornwall and in the Padstow area.

**Conclusions:**

This study shows marked geographical differences in allergy referrals which are likely to reflect a combination of environmental factors and GP referral patterns. The data suggest that GPs may benefit from education and ongoing decision support and be supported by public education on the nature of allergy. It suggests further research into what happens to patients with allergy where there has been low use of tertiary services and further research into cross-reactivity and co-occurrence, and spatial distribution of allergy.

## Background

The predominant allergies seen in allergy practices are type I hypersensitivity (IgE mediated) reactions. These occur rapidly, with typical symptoms (urticaria, angioedema, conjunctivitis, rhinitis, wheezing or anaphylaxis), have a well-defined mechanism and have validated tests to confirm the diagnosis. Non toxic adverse reaction to food can be a result of food allergy or food intolerance [1]. Food allergies can be either IgE mediated (as above) or non-IgE mediated (usually with delayed onset, gastrointestinal or non-specific symptoms, where the mechanism is unclear and are harder to diagnose with no validated tests). Lack [1] reported that IgE-mediated food allergies affect between 6 and 8% of children in the United Kingdom and the United States. Although up to 25% of adults report symptoms that may be related to certain foods, the prevalence of food allergies among adults is less than 3%. Nevertheless, admissions in English NHS hospitals for (all cause) anaphylaxis (severe allergic reactions) rose steadily from 2821 in 2004-05 to 3595 in 2008-09 [2]. Anaphylaxis is responsible for the death of approximately 1 to 3 individuals per million population [3].

In 2006 the Department of Health published a review of Allergy Services [4]. They recommended action to improve services and pointed to the need to: (i) establish levels of need for services for allergy, (ii) explore the scope for creating additional training places for allergists, (iii) consider the development of guidance for referral and care pathways. In 2010, a joint Royal College of Physicians and Royal College of Pathologists Working Party surveyed respiratory physicians, immunologists, allergists, and others and made recommendations about the organisation of allergy services [5]. However, relatively little has been published on the effectiveness and efficiency of the referral process to tertiary centres.

Studies from a number of European countries have shown evidence of geographical differences in allergy prevalence [6-16]. Meta-analyses have confirmed a marked heterogeneity in the prevalence of food allergy that could be a result of real differences between populations or differences in study design or methodology [17,18].

The Peninsula Allergy Service (PAS) was set up in 1996 at Derriford Hospital, Plymouth, serving Devon and Cornwall in the UK. Initially, patients of all ages (including children) were seen, but from 2004, children were normally referred to a paediatric clinic. The SW Peninsula of Devon and Cornwall is a largely rural area with population (2001) of 1.6 million. Travel from the far south west to Plymouth can be time consuming and expensive (Land's End to Plymouth is a 2-3 hour 115-mile trip each way by car). The shape of this region, bounded by the sea, means that referral to any other tertiary allergy service is extremely unlikely; our audit of referrals was therefore for a geographically defined population.

The aim of this study was to audit eleven years of patients referred to the PAS. Amongst those diagnosed as having an allergy, we aimed to explore: (i) differences over time and by demographics, (ii) the types of allergy, (iii) co-occurrence, and (iv) spatial distributions to identify areas with high or low referral of true cases.

## Methods

### Ethics

Ethic approval was not required as this was purely an audit project using anonymised patient data

### Data Sources

The main source of data was consultant letters to GPs summarising the diagnosis of patients, archived from September 1998 to September 2009. These letters were for patients who, following the taking of a detailed history, were confirmed as having an allergy (type I hypersensitivity) on the basis of a 'positive' skin prick test or specific IgE test in the clinic. A skin prick test was considered positive when the diameter of the wheal exceeded the diameter of the negative control by 3 mm and when a flare reaction was also present. A specific IgE test was considered positive when the value exceeded 0.7 kUA/l. We did not include borderline positive results as positive. Letters were reviewed, extracting the clinic date, doctor seen, patient's name, gender, date of birth, postcode, GP, and diagnoses. There were 1076 visits (letters to GPs). Ninety patients had more than one consultation; we deleted 111 subsequent consultations just keeping the earliest consultation for 965 individuals. Four patients had postcodes that were out of area (from armed forces, Somerset, and Bristol). Further analysis was on anonymised data for 961 patients.

Supplementary sources of data were: (1) New referral statistics to the Peninsula Allergy Service extracted from the hospital information system (used to assess the proportion of referrals who were diagnosed as having an allergy); (2) Population data by postcode, aggregated from 2001 census data from the Office of National Statistics, used as the denominator to calculate rates by postcode area; (3) Deprivation decile data by postcode for England, from Dr Iain Paterson, University of Glasgow, used in one cross-tabulation to see if the referred and diagnosed patients were equally likely to be from different deprivation deciles.

### Aggregation and 'cleaning' of allergy diagnoses

The original allergy diagnoses were kept as part of the database but diagnoses were recoded and aggregated based on common characteristics of the allergen such as method of entry to the body (e.g. airborne Vs food), source (e.g. the sea), and common English usage on categories (e.g. nuts).

### Non spatial analysis

We used simple descriptive analyses to describe types of allergy and differences over time, by age at referral, distributions by GP and practice location. For example, we investigated co-occurrence of (non-aggregated) food allergies by 21 cross-tabulations and Fisher exact test highlighting those where a Fisher exact test gave p < 0.024, that is applying a Bonferroni correction to set a level where co-occurrence may be more than expected by chance. More details of analysis are presented in Additional File 1.

### Spatial analysis

There may be a number of underlying reasons why there are differences in the spatial distribution of allergies referred to, and diagnosed at, a regional allergy service, including: (1) unmet need, i.e. people with allergies who may not have been diagnosed, (2) alternative referral or treatment, i.e. patients who are either diagnosed and treated by the GP or referred to another hospital clinic, (3) 'real' spatial differences perhaps due to environmental influences. To explore this we used postcodes for each patients (numerator), postcode district boundary data from Edina http://edina.ac.uk/digimap/index.shtml, and denominator populations from the 2001 Census. There were 109 postcode districts in the four postcode areas of TQ (Torquay), EX (Exeter), PL (Plymouth), TR (Truro) covering Devon and Cornwall. We calculated the 11 year standardised morbidity ratio for allergy referrals in each postcode district by comparing the observed count of referrals with the expected count using simple internal standardisation based on total numbers of referrals for the Peninsula and total population count in each district. It is standard to assume that the observed counts follow a Poisson distribution. We used three methods to determine whether this assumption was consistent with the data: (i) a χ^2 ^test for overdispersion, (ii) Potthof-Whittinghill's test of overdispersion [19], and (iii) Moran's I test of spatial autocorrelation [20]. We present three maps: (i) 'crude' 11 year rates (number of cases/postcode population), (ii) 'smoothed' maps which try to allow for the small numbers in each postcode district by averaging uncertainty across neighbouring districts, and (iii) 'difference' maps which try to remove the 'referral effect' that might be due partly to distance from Derriford Hospital and GP propensity to refer, leaving any possible environmental effect. More details of the statistical analysis used in the spatial analysis and mapping are given in Additional File 2.

## Results

### Allergy cases as a proportion of all referrals

Table 1 presents data from 'counts' of referrals combined with data of confirmed cases to show the proportion of all referrals that were confirmed as having a 'true allergy'. Only 1 in 5 patients (12-23%) were diagnosed as having an allergy, with the majority of other referrals consisting of patients with idiopathic urticaria, food intolerance or non-specific symptomatology.

**Table 1:** Referrals to the Peninsula Allergy Service and the number of confirmed cases.

Year	Referrals	Confirmed	Percentage of referrals
1998	287	6	Part year
1999	335	73	22
2000	459	107	23
2001	414	92	22
2002	477	75	16
2003	438	71	16
2004	415	48	12
2005	457	71	16
2006	498	99	20
2007	613	98	16
2008	598	107	18
2009	787	114	Part year

**TOTAL**	5778	961	18*

* the number of referrals was only available for full years so percentages of confirmed cases is only shown for ten full years and the overall percentage for those ten year.

### Age and gender of patients

The mean age of patients at referral was 35 (median 33). There were considerably more females than males (336 M (35%), 625F (65%)). There was no difference in mean age between males and females. Excluding 96 children, the mean age was 36 for women and 39 for men.

### Diagnosis rate by year and area

The number referred and diagnosed with allergy each month ranged from 0 to 21. Initially (1998-2001) referrals were predominantly from Plymouth postcodes but the percentage coming from other postcode areas increased to 40% in 2002-2005 and just under half in 2006-2009 (Additional file 1, Table A1).

### Referring GPs

The 961 patients with diagnosed allergy were referred by a total of 672 different GPs. Most GPs (509, 76%) referred only one patient. The most patients referred by any one GP was 11. The 672 GPs came from 190 practices with the maximum referred from one practice being 39. Although this may reflect the presence of one or two very large practices, the variations between practices were considerable especially in Truro and Exeter postcodes. Of the 28 practices with Truro postcodes one practice referred 27 people while the next most frequent referred only five. Of 44 practices with Exeter postcodes one practice referred 25 with the next most frequent referring only seven.

### Types of allergy

**Figure 1:**
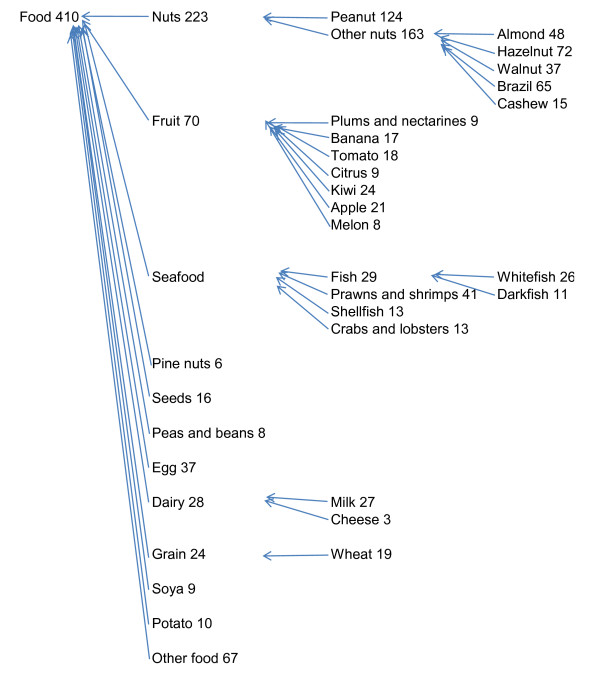
**Hierarchy of food allergies for 961 people referred and diagnosed over 11 years at the Peninsula Allergy Service**.

**Figure 2:**
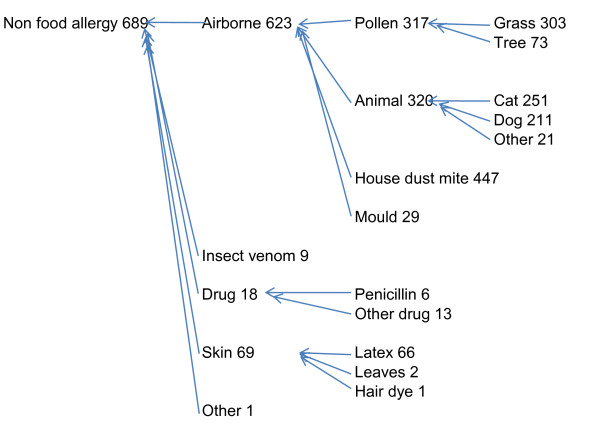
**Hierarchy of non-food allergies for 961 people referred and diagnosed over 11 years at the Peninsula Allergy Service**.

Figures 1 and 2 show that 410/961 (43%) had one or more food allergies and 689/961 (72%) had one or more non-food allergies; 15% (142) had both food and non-food allergy. Nuts, fruit, and seafood (including fish and crustaceans), were the most frequent food allergies. Of the non food allergies, airborne allergies were the most frequent 623/689 and of these house dust mite, pollen, and animal hair all occurred frequently. The gender difference (more females than males) was greater for non food allergies (466F to 223M/689) than for food allergies (260F to 150M/410).

### Co-occurrence of allergies

We examined the co-occurrence of allergies, and used knowledge of cross-reactivity together with other allergen characteristics to aggregate and create 'allergy hierarchies' (Figures 1 and 2). For example, peanut, almond, hazel nut, walnut, brazil nut, cashew nut allergies co-occurred frequently. Pine nuts did not co-occur with these other nuts and but more often with seed allergies, but we have left them as separate items in most analyses. There was co-occurrence of white fish and dark fish allergies, and between (white and dark) fish and shellfish, and between fish and prawn-shrimp. Although fish did not co-occur so frequently with crab-lobster, as crab-lobster co-occurred with prawn-shrimp, we aggregated all into 'seafood'. In some cases, numbers were small and we grouped using common 'English language' categories, so only six people had penicillin allergies of which one was also allergic to 'other drugs' (erythromycin) but we nevertheless grouped as 'drugs'.

There were very high rates of co-occurrence of pollen, house dust mite, and animal hair allergies, so these were grouped as 'airborne' allergies. Table 2 shows the chances of being allergic to pollen, house dust mite, or animal hair given the known allergy to one of these three. For example, of those allergic to house dust mite, 53% were also allergic to animal hair and 46% to pollen. Of those allergic to pollen, 65% were allergic to house dust mite and 50% to animal hair.

**Table 2:** Given known allergy to first allergen, the proportion who are allergic to second allergen.

		Second Allergen			
		House dust	Animal	Pollen	N
**First Allergen**	House dust Animal Pollen	74% 65%	53% 50%	46% 49%	447 320 317

### Other co-occurring allergies

There were other co-occurring food allergies that were not aggregated on the basis of common English language aggregate terms but are interesting to note as possible cross-reactivity or co-occurrence for other reasons. These are shown in Table 3. Of the 28 people allergic to dairy products, 13 were also allergic to egg, and seemed to have slightly more chance of allergy to other foods. Of 66 people allergic to latex 15 were also allergic to fruit. As previous authors had found cross-reactivity between latex and food allergies we also explored this further. Six out of 17 people allergic to banana and 5 out of 24 allergic to kiwi were also allergic to latex. There did not appear any association with other types of fruit or other foods.

**Table 3:** Co-occurring food allergies amongst 961 patients, showing most frequent food groups and the observed/expected numbers of patients with each allergy from 2 × 2 cross-tabulations.

	nuts	sea	dairy	fruit	egg	grain	latex
nuts	N = 223						
seafood	10/16.5	N = 71					
dairy	8/6.5	6/2.1	N = 28				
fruit	22/16.2	6/5.2	7/2.0	N = 70			
egg	15/8.6	6/2.7	**13/1.1**	6/2.7	N = 37		
grain	6/5.6	2/1.8	3/0.7	3/1.7	4/0.9	N = 24	
latex	7/15.3	5/4.9	6/1.9	**15/4.8**	5/2.5	3/1.6	N = 66

The two bold cells (egg-dairy) and (latex-fruit) show where there is more co-occurrence than you would expect by chance (based on Fisher exact test and p < 0.0024).

### Number of allergies and unusual cases

Most patients (60%) had more than one allergy (median two) recorded. One patient had 10 items, one 11 items, and one 22 items (House dust mite, aspergillus, milk, wheat, egg, honey bee, grass pollen, peanuts, mixed fish, latex, tomatoes, raisins, rapeseed, lemon, grape, spices, carrots, asparagus, orange, pork, chicken and potato). Some of the rarer allergies included seminal fluid, celery, various medications (including porcine and human recombinant insulin, Hydroxocobalamin, and Fluoxetine), goat, yoghurt, and Pudu.

### Changes over time

Although the number of patients referred and diagnosed each year has increased, this seems to be due to an increase in all allergies and not any particular allergy. (Additional file 1, Table A2 and Additional file 1, Figure A1).

### Age-gender differences in allergies

People referred with food allergies were on average 5 years younger than the other referrals (37 Vs 32; t = 5.1; p = 0.017). Amongst the 410 with food allergy, those allergic to seafood were older (39 Vs 30; t = -4.4; p < 0.001), those allergic to nuts were much younger (26 Vs 38; t = 8.7; p < 0.001), those to allergic to egg were younger (25 Vs 32; t = 2.7; p = 0.008), but there was no age difference for fruit, dairy, or grain. The children under 16 were much more likely to have nut allergy (45% Vs 21%, χ^2 ^= 28, p < 0.001) than adults. The only gender difference was for fruit: females were more likely to have fruit allergy (20 Vs 11%; χ^2 ^= 5.5; p = 0.019).

### Deprivation categories

There was no significant difference in the deprivation scores of the 961 compared to all postcodes for Devon and Cornwall (Additional file 1, Table A3). Analysis of individual allergy types did not show any significant differences by deprivation.

### Geographic distribution of allergies

**Figure 3:**
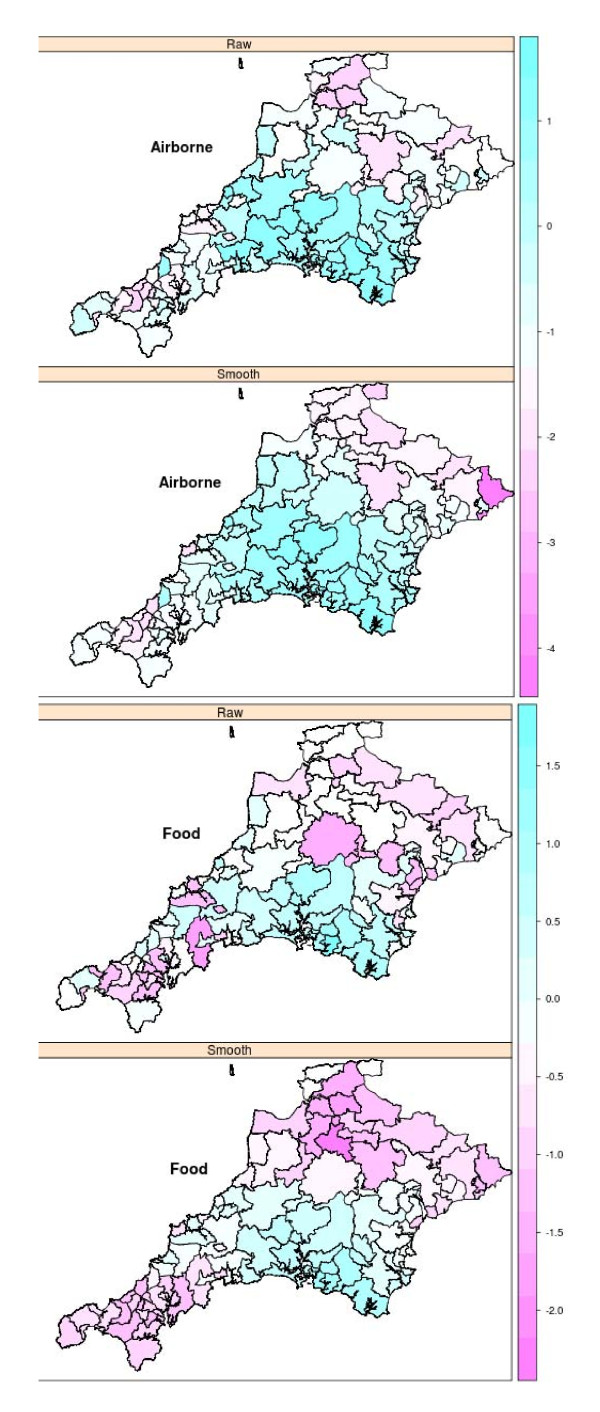
**Postcode district maps showing Log of Standardised Morbidity Ratios for Allergies (as referred to Derriford Hospital), crude rates and smoothed rates: Airborne (n = 623), Food (n = 410)**.

Analysis of the 11- year rates by postcode district showed that there is evidence for a non-homogenous spatial distribution. This was also true if you looked at the subgroups of airborne allergies, pollen allergies, food allergies, and nut allergies. There was no clear evidence of spatial structuring for seafood or fruit, though in both cases the number of cases was small (n = 71 and 70). However, latex allergies, also with a small sample (n = 66), did have evidence of spatial structuring. Figure 3 shows crude and smoothed rates for airborne and food allergies. There appears to be a clear referral effect, i.e. some form of clustering around the referral hospital. However, the geographical distribution appears to vary between allergies, so for example, airborne (particularly pollen allergies) seem to cluster more to the north of Plymouth, whereas food allergies (particularly nut allergies) cluster more in the South Hams (to the South East of Derriford Hospital). Additional File shows other maps of Pollen (n = 317), Nuts (n = 223), Fruit (n = 70), Seafood (n = 71), Latex (n = 66)). Seafood, although having small numbers and not statistically significant in the analysis above, seems to show a different distribution perhaps related to distance from the sea.

**Figure 4:**
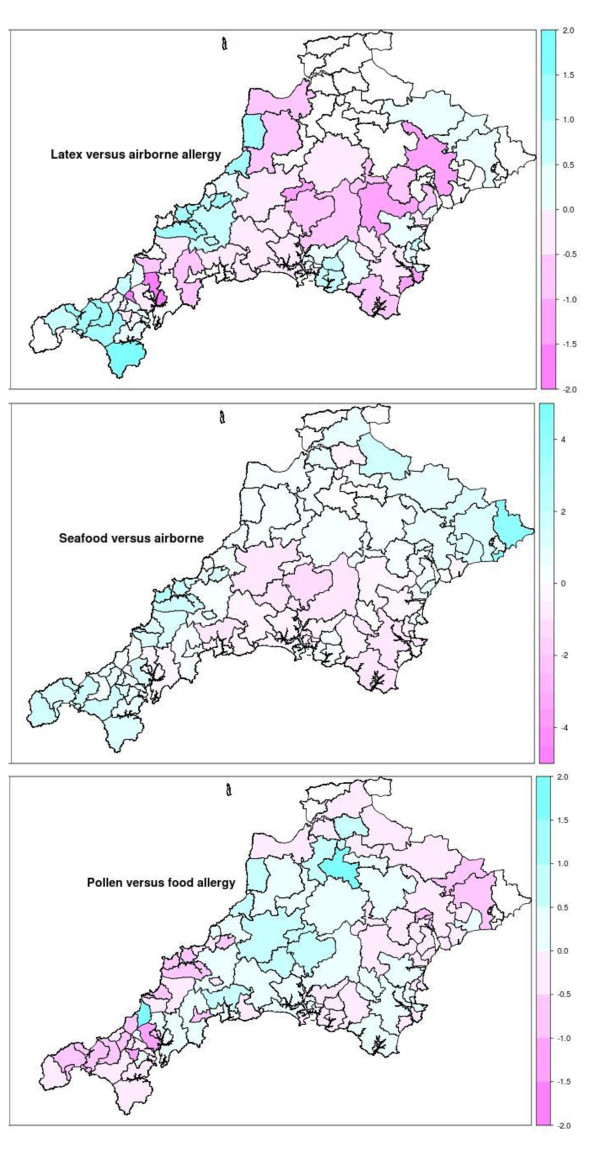
**Postcode district maps showing Log of Standardised Morbidity Ratios for Allergies (as referred to Derriford Hospital), comparison of Pollen versus Food, Latex versus Airborne, Seafood versus Airborne**.

Figure 4 shows differences between particular allergies and a 'control' allergy to try to 'remove' the referral effect and explore possible environmental effects. We can see that comparing pollen allergy with food allergy we see more pollen allergies in the North Dartmoor and Exmoor areas. Comparing seafood with airborne, we see more seafood allergies in the far south west of Cornwall and in the Padstow area.

## Discussion

It is difficult to compare the 11 year incidence rate of confirmed cases referred to this regional allergy service with previous research. Given the clinical confirmation of their allergies our population would have very few, if any, 'false positives'. However, given the variability of referral by GPs, our incidence rate at a regional service would grossly underestimate the true population prevalence. On the other hand, many publications are population self-report 'allergies' [8,9,11,13] many of which are likely to be due to intolerance or other conditions rather than allergies. Our cases were not self-report. Although both patients and GPs may have initially thought they 'had an allergy', resulting in the referral to PAS, at best, only 23% of any one year's referrals were found to be allergy. There may be misconceptions about allergy in the public [21]. From clinical experience, many individuals attribute a number of non-specific symptoms, particularly gastrointestinal, headaches, etc to allergies. In addition to this, certain symptoms such as urticaria or angioedema which are indeed associated with allergies, can also occur due to non-allergy causes, and may be 'idiopathic' [22,23].

GPs may come under pressure from the general public for referral and an expert opinion [24]. Based on 800 new patients per year to the PAS, the cost to Primary Care Trusts in Devon and Cornwall is approximately £300,000 (based on local cost data). Only one in five of these patients have an allergy. Previous unpublished audits suggest that at least a quarter of the referrals could be avoided saving £75,000 a year for the NHS and unnecessary travel and time off work for the patient.

There are few estimates of referred and confirmed allergy derived from clinic based studies. Asero et al [15] reported the types of allergy found in 25601 patients attending 17 allergy clinics throughout Italy, and Joral et al [6] reported the numbers of patients with food allergies in 3034 patients referred to two hospitals in the Basque country, but neither attempted to estimate population prevalence. A prevalence study in Madrid estimated that 11.6% of patients attending general practice had some allergy [25].

In a multicentre study in 1991-94, Woods et al found that 12% of adults aged 20-44 from 15 countries, reported (on interview) adverse reactions to particular foodstuffs. This self-report figure varied considerably from 4.6% in Spain to 19.1% in Australia [26]. The true diagnosis of food allergy requires double blind, placebo-controlled food challenges (DBPCFC). In two DBPCFC population studies in the mid 1990 the prevalence of food allergy was estimated at 2.4% in the Netherlands [27] and 1.4--1.8% in the UK [28]. In this audit of a clinic population, 410 cases of food allergy were referred to the PAS over 11 years from a population of 1.6 million which is 0.03% or a factor of 100 less than reported in these population studies. This suggests that very few people with allergy present to their GP and subsequently get referred.

Spatial structuring of rates of confirmed allergy referred to the PAS is quite clear. There is a clear proximity or referral effect, i.e. more referral from GPs closer to the hospital. We do not know what happens to people with allergies in more remote areas. In addition, assuming the proximity effect is consistent across different types of allergy, comparisons between allergies suggest environmental influences.

This audit therefore suggests that there are many people with allergy who could benefit from referral. The cost and impact of undiagnosed and untreated allergy can be considerable [4]. Based on 2009 data, if Exeter, Torquay, and Truro postcodes (i.e. those more remote from this regional centre) had the same levels of referral of true cases as Plymouth postcodes, then the number of true cases referred would increase from 150 to 222 per year. That there may be environmental influences on the prevalence of allergy is not new. For example, in Guipuzcoa in Spain, cow's milk and eggs were infrequent and fruit, seafood and vegetables were common allergies [6]. Joral et al attributed this to dietary habits. Two recent large studies appear to give contradictory evidence on air pollution and respiratory allergies. A large American study found evidence of adverse health for children living in areas with chronic exposure to higher levels of ozone and particulate matter with diameter less than 2.5 micrometres compared with children with lower exposure [29]. On the other hand a large UK study found no relationship between self-reported wheezing in the past year, asthma, eczema and hay fever and living within 150 m from a major road [30].

We are not aware of any previous spatial analysis based on referred and confirmed cases of allergy in a defined region such as the South West Peninsula. Pollen allergies seemed more prevalent in moor areas, food allergies (particularly nut allergies) in the South Hams, and skin allergies around Derriford Hospital. On small numbers, there was some indication of food from the sea in the far south west of Cornwall and in the Padstow area. These are based purely on an 'ecological association' and we have no evidence that these individuals had more contact with their allergens.

Cross-reactivity between allergens has been described before but usually for food types which are taxonomically similar. For example, Lack [1] summarises findings showing cross-reactivity between peanuts and legumes, peas, lentils and co-reactivity with tree nuts. Allergies in children are commonly directed to proteins from eggs, milk, wheat or soy [31]. Other recognised cross-reactivity syndromes include the latex fruit syndrome (cross reactivity between latex and avocados, bananas, kiwi, etc) [32] and the birch-fruit syndrome (cross reactivity between Birch pollen and a number of fruits such as apple, peach, plum and hazelnuts) [33]. In France in 2001, Kanny found that food allergy was four times more likely in people with allergy to latex [11]. We found no such association with food (perhaps reflecting different levels of fruit consumption in France and SW England), but there were co-occurrence of latex with banana and kiwi allergies. Our data showed clear relationships between egg and dairy allergies and some suggestion (based on small numbers) of other co-occurrence that would be worthy of further investigation.

Our audit has limitations from combining different data sources. Our estimates of true positive/all cases are based on the numerator from letters from the PAS to GPs about confirmed cases while the denominator comes from hospital statistics of referral. This means we cannot separate the true positive/all cases between inhalant and food allergy as we do not have separate denominators. Our spatial analysis uses population figures from the 2001 census and it is possible that some areas may have had population migration affecting referral rates.

## Conclusions

This 11-year audit of a regional service serving a well-defined geographical area suggests that there is both unmet need and inappropriate referral. GPs may benefit from education and ongoing decision support. Public education on the nature of allergy, and intolerance and how these may be treated should also be considered. It also suggests further research into what happens to patients with allergy where there has been low use of tertiary services. Finally, it suggests further research into co-occurrence, cross-reactivity, and spatial distribution of allergy.

## List of Abbreviations

GP: General Practitioner; IgE: Immunoglobulin E (see e.g. http://en.wikipedia.org/wiki/Immunoglobulin_E); NHS: National Health Service; PAS: Peninsula Allergy Service.

## Competing interests

The authors declare that they have no competing interests.

## Authors' contributions

ERK and RBJ had the idea for the study. RBJ managed the project, carried out initial data cleaning and analysis including initial spatial analysis, and wrote the paper. PH carried out the definitive spatial analysis and edited the paper. ERK was lead clinician in the PAS and was responsible for the clinical data used, with RBJ oversaw the data entry and cleaning, added clinical context to the paper and advised RBJ on aspects of analysis, worked with RBJ on the literature review, and edited the paper. All authors read and approved the final manuscript.

## Pre-publication history

The pre-publication history for this paper can be accessed here:

http://www.biomedcentral.com/1471-2458/10/790/prepub

## Supplementary Material

Additional file 1 **Table A1**. Numbers referred and diagnosed with allergy by year of clinic and 2 character postcode of address, showing also the total population from 2001 census and 11 year rate per 100,000 population. Table A2. Number of diagnoses for food and non food allergies showing most frequently occurring sub groups, by year (partial years in 1998 and 2009). Table A3. 'Deprivation deciles of the 961 people in this study, postcodes in Devon and Cornwall, and in England and Wales. Figure A1. Number of confirmed cases referred by year and type of allergy. Figures A2. Log of Standardised Morbidity Ratios for Allergies (as referred to Derriford Hospital), crude rates and smoothed rates: Pollen (n = 317), Nuts (n = 223), Fruit (n = 70), Seafood (n = 71), Latex (n = 66). Click here for file

Additional file 2 **Further details of the spatial analysis**. Click here for file
