# Supplementary material for: Referrals to a regional allergy clinic - an eleven year audit
Source: BMC Public Health. 2010 Dec 29;10:790. doi: 10.1186/1471-2458-10-790 (PMC3022859; doi:10.1186/1471-2458-10-790)
Supplement: Additional file 1 — Table A1. Numbers referred and diagnosed with allergy by year of clinic and 2 character postcode of address, showing also the total population from 2001 census and 11 year rate per 100,000 population. Table A2. Number of diagnoses for food and non food allergies showing most frequently occurring sub groups, by year (partial years in 1998 and 2009). Table A3. 'Deprivation deciles of the 961 people in this study, postcodes in Devon and Cornwall, and in England and Wales. Figure A1. Number of confirmed cases referred by year and type of allergy. Figures A2. Log of Standardised Morbidity Ratios for Allergies (as referred to Derriford Hospital), crude rates and smoothed rates: Pollen (n = 317), Nuts (n = 223), Fruit (n = 70), Seafood (n = 71), Latex (n = 66). [file 1471-2458-10-790-S1.DOC]

**Additional File 1**

**Appendix: Extra tables**

| **Year of clinic** | **Two character postcode** | | | |  |
| --- | --- | --- | --- | --- | --- |
|  | **EX**  **(Exeter)** | **PL (Plymouth)** | **TQ**  **(Torquay)** | **TR**  **(Truro)** | **Total** |
| 1998 (from Sep) | 0 | 6 | 0 | 0 | 6 |
| 1999 | 4 | 59 | 9 | 1 | 73 |
| 2000 | 6 | 72 | 24 | 5 | 107 |
| 2001 | 5 | 65 | 18 | 4 | 92 |
| 2002 | 1 | 45 | 28 | 1 | 75 |
| 2003 | 8 | 47 | 12 | 4 | 71 |
| 2004 | 4 | 30 | 9 | 5 | 48 |
| 2005 | 6 | 47 | 17 | 1 | 71 |
| 2006 | 8 | 56 | 22 | 13 | 99 |
| 2007 | 22 | 45 | 18 | 13 | 98 |
| 2008 | 24 | 57 | 14 | 12 | 107 |
| 2009 (to Sep) | 28 | 54 | 22 | 10 | 114 |
| Total | 116 | 583 | 193 | 69 | 961 |
|  | | | | | |
| 2001 pop | 509911 | 510223 | 276562 | 277222 | 1573918 |
| 11 year rate per 100,000 | 23 | 114 | 70 | 25 | 61 |

Table A1. Numbers referred and diagnosed with allergy by year of clinic and 2 character postcode of address, showing also the total population from 2001 census and 11 year rate per 100,000 population.

| Year of clinic | Non food | | | | Food | | | | All people |
| --- | --- | --- | --- | --- | --- | --- | --- | --- | --- |
|  |  | Pollen | Dust Mites | Animal Hair |  | Nuts | Seafood | Fruit |  |
| 1998 (3m) | 2 | 0 | 1 | 1 | 4 | 2 | 0 | 1 | 6 |
| 1999 | 49 | 15 | 36 | 28 | 32 | 21 | 2 | 4 | 73 |
| 2000 | 86 | 34 | 64 | 46 | 42 | 28 | 3 | 5 | 107 |
| 2001 | 67 | 15 | 43 | 36 | 40 | 24 | 5 | 8 | 92 |
| 2002 | 62 | 36 | 40 | 31 | 32 | 12 | 6 | 9 | 75 |
| 2003 | 45 | 19 | 35 | 19 | 36 | 22 | 7 | 5 | 71 |
| 2004 | 35 | 12 | 18 | 16 | 23 | 13 | 3 | 2 | 48 |
| 2005 | 54 | 25 | 34 | 26 | 28 | 16 | 4 | 4 | 71 |
| 2006 | 65 | 27 | 40 | 26 | 44 | 20 | 8 | 9 | 99 |
| 2007 | 67 | 41 | 43 | 30 | 43 | 21 | 8 | 8 | 98 |
| 2008 | 77 | 42 | 51 | 26 | 38 | 23 | 13 | 6 | 107 |
| 2009 (9m) | 80 | 51 | 42 | 35 | 48 | 21 | 12 | 9 | 114 |
| **All** | **689** | **317** | **447** | **320** | **410** | **223** | **71** | **70** | 961 |

Table A2. Number of diagnoses for food and non food allergies showing most frequently occurring sub groups, by year (partial years in 1998 and 2009).

| Dep’n  Decile | Allergy cases this study | |  | All postcodes   In Devon and Cornwall | |  | All postcodes in England and Wales | |
| --- | --- | --- | --- | --- | --- | --- | --- | --- |
|  |  |
|  | n | % |  | n | % |  | n | % |
| 1 | 41 | 4.3 |  | 3269 | 5.2 |  | 239831 | 14.5 |
| 2 | 142 | 14.8 |  | 9708 | 15.4 |  | 202136 | 12.2 |
| 3 | 163 | 17.0 |  | 13318 | 21.1 |  | 176600 | 10.6 |
| 4 | 203 | 21.1 |  | 11654 | 18.4 |  | 162342 | 9.8 |
| 5 | 149 | 15.5 |  | 10871 | 17.2 |  | 159129 | 9.6 |
| 6 | 125 | 13.0 |  | 7522 | 11.9 |  | 148721 | 9.0 |
| 7 | 86 | 8.9 |  | 4028 | 6.4 |  | 141897 | 8.6 |
| 8 | 48 | 5.0 |  | 2219 | 3.5 |  | 132979 | 8.0 |
| 9 | 4 | .4 |  | 418 | .7 |  | 138669 | 8.4 |
| 10 | 0 | 0 |  | 89 | .1 |  | 150282 | 9.1 |
| Total | 961 | 100.0 |  | 63096 | 99.9 |  | 1652586 | 99.6 |
| Missing | 0 | 100 |  | 94 | .1 |  | 6354 | .4 |

Table A3. ‘Deprivation deciles of the 961 people in this study, postcodes in Devon and Cornwall, and in England and Wales.

Figure A1.

Number of confirmed cases referred by year and type of allergy.

| Figures A2. Log of Standardised Morbidity Ratios for Allergies (as referred to Derriford Hospital), crude rates and smoothed rates: Pollen (n=317), Nuts (n=223), Fruit (n=70), Seafood (n=71), Latex (n=66). | 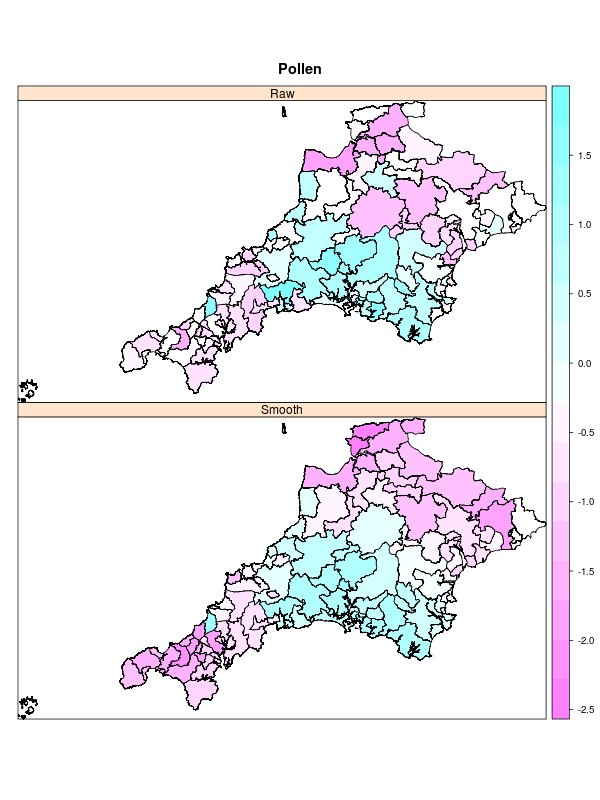 |
| --- | --- |
| 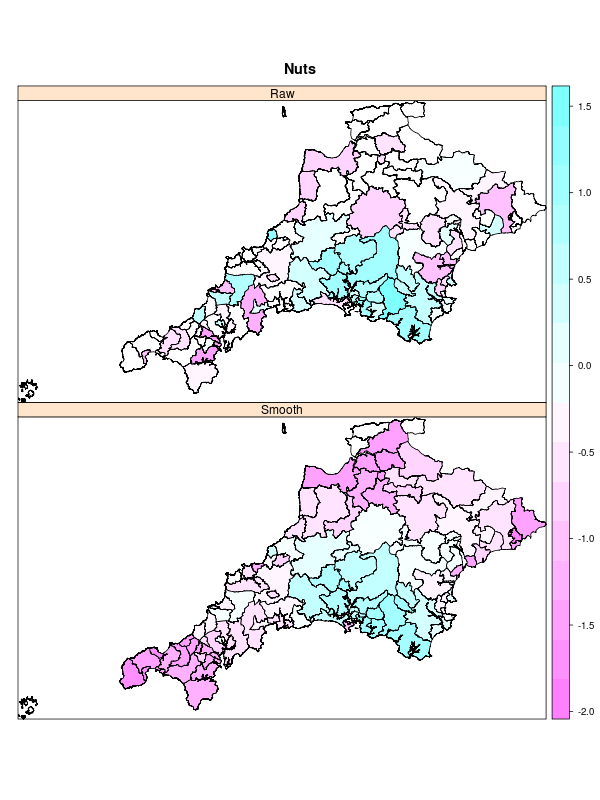 | 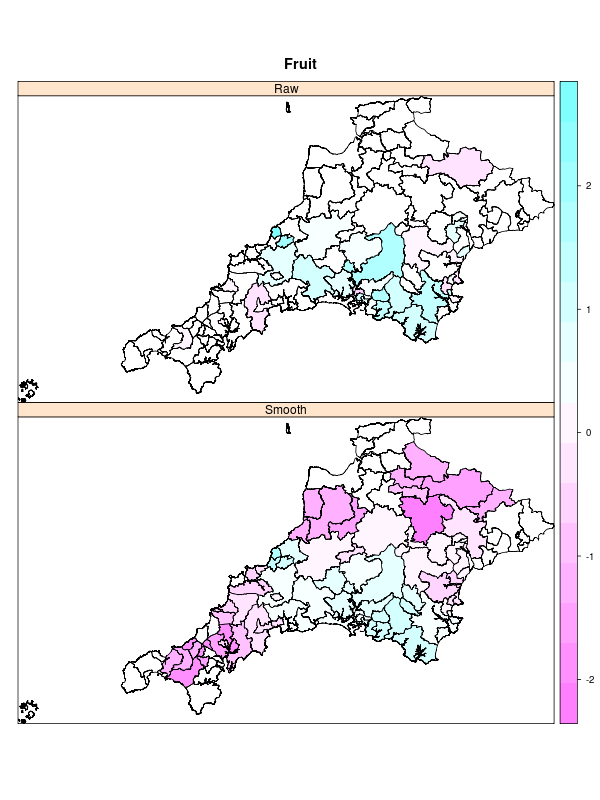 |
| 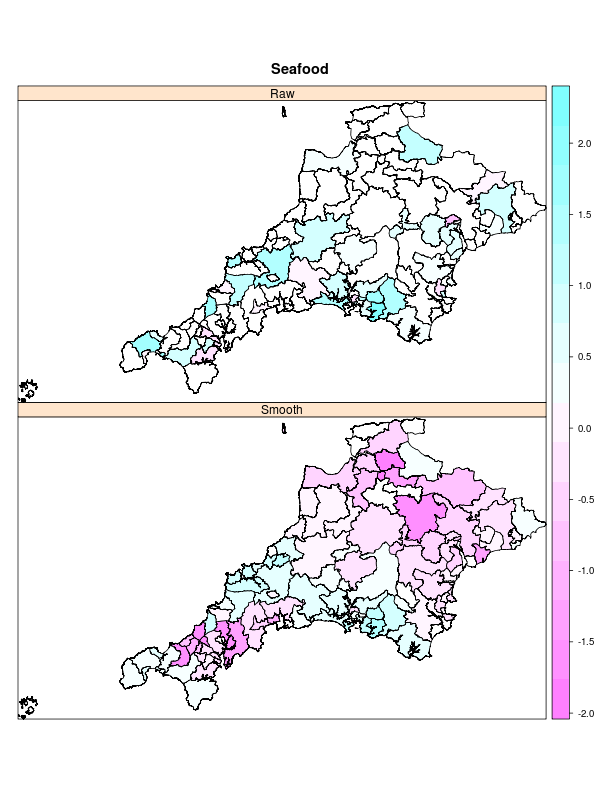 | 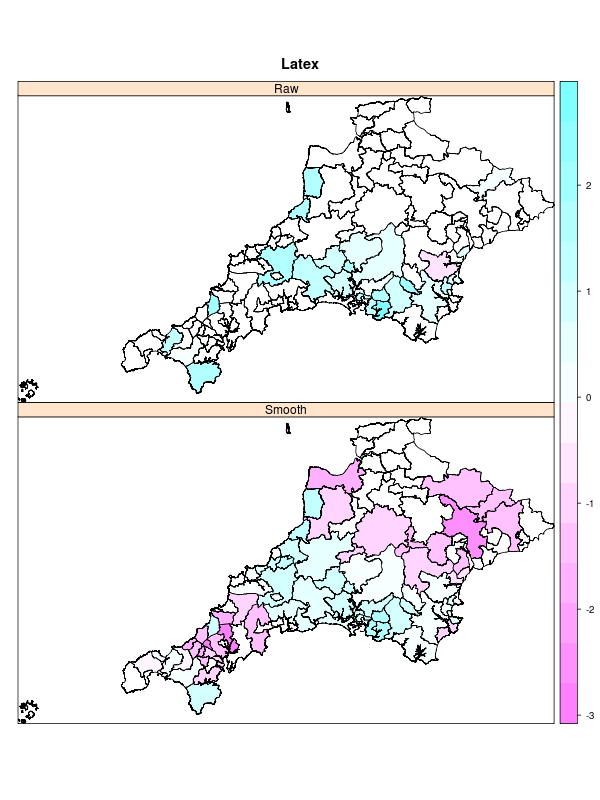 |
