# Supplementary material for: Referrals to a regional allergy clinic - an eleven year audit
Source: BMC Public Health. 2010 Dec 29;10:790. doi: 10.1186/1471-2458-10-790 (PMC3022859; doi:10.1186/1471-2458-10-790)
Supplement: Additional file 2 — Further details of the spatial analysis. [file 1471-2458-10-790-S2.PDF]

Explanation of analysis of the Allergy Data

June 2, 2010

1 Statistical Appendix

In total, 961 cases were reported in the time period.

	n
Airborne	623
Food	410
Pollen	318
Seafood	71
Nuts	223
Fruit	70
Latex	66

1.1 Disease Rates

The data were investigated using conventional epidemiological methods, e,g, Lawson (2001). Denote the $p = 7$ allergy types by $j = 1, \ldots, 7$ (Airborne, Food, Pollen, Seafood, Nuts, Fruit, Latex), and the $n = 109$ postcode sectors by $i = 1, \ldots, 109$.

As is conventional, the population in postcode sector i is denoted P_i and the number of cases of allergy type j in area i is denoted O_{ij}. Indirect standardisation was used to calculate the expected number of cases in each postcode sector. To do this, an overall incidence rate for the peninsula was calculated for each allergy as

$$r_j = \frac{\sum_{i=1}^{n} O_{ij}}{\sum_{i=1}^{n} P_i}$$

and then the expected allergy count for allergy j can be calculated as:

$$E_{ij} = r_j P_i$$

The basic estimate for the risk of each allergy j in each postcode sector i is therefore esimated by the Standardised Morbidity Ratio (SMR):

$$SMR_{ij} = \frac{O_{ij}}{E_{ij}}$$

Results may be presented in terms of this SMR or the $\log(SMR)$.

In modelling the SMR we assume that O_{ij} is Poisson distributed such that:

$$O_{ij} \sim Poisson(E_{ij}\lambda_{ij}) \tag{1}$$

and model the unknown parameter λ_{ij} in order to understand the risk surface.

Analysis of these data was performed using the R software (R Development Core Team, 2009).

1.2 Confidence intervals for the relative risk parameter

We first examine methods for calculating the exact 95% confidence intervals proposed by Day (1992) and implemented by Aragon (2009). These are displayed in the form of caterpillar plots.

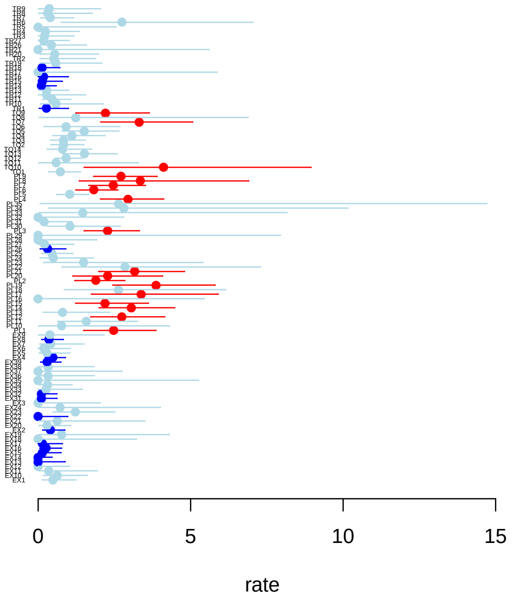

Figure 1: 95% Confidence interval for log relative risk parameter λ_{ij} for Airborne allergens (four postcode sectors omitted due to very small population), those entirely above one highlighted in red, those entirely below one highlighted in dark blue

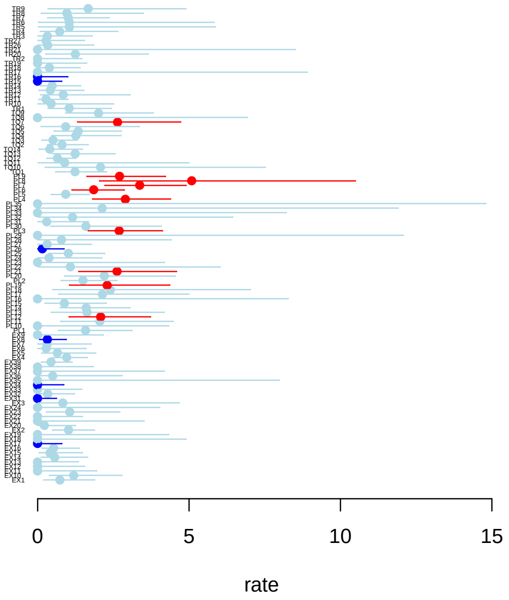

Figure 2: 95% Confidence interval for log relative risk parameter λ_{ij} for Food allergies (four postcode sectors omitted due to very small population), those entirely above one highlighted in red, those entirely below one highlighted in dark blue

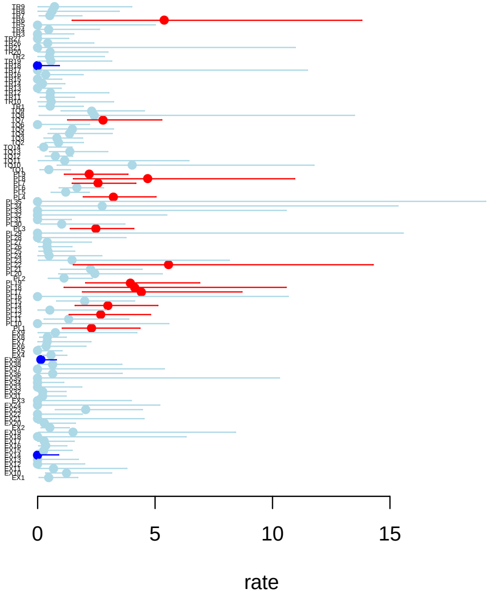

Figure 3: 95% Confidence interval for log relative risk parameter λ_{ij} for Pollen allergies (four postcode sectors omitted due to very small population), those entirely above one highlighted in red, those entirely below one highlighted in dark blue

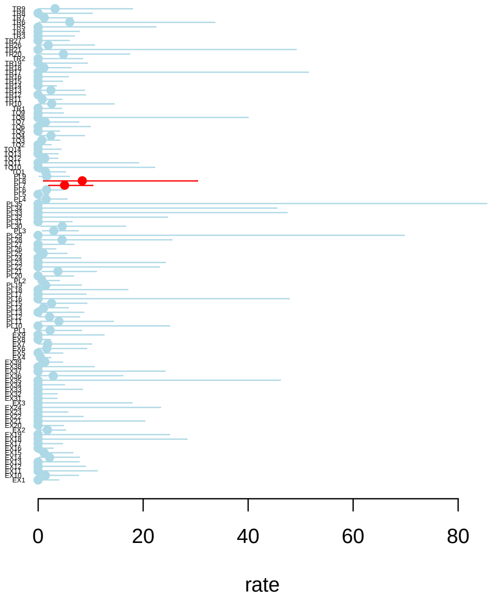

Figure 4: 95% Confidence interval for log relative risk parameter λ_{ij} for Seafood allergies (four postcode sectors omitted due to very small population), those entirely above one highlighted in red, those entirely below one highlighted in dark blue

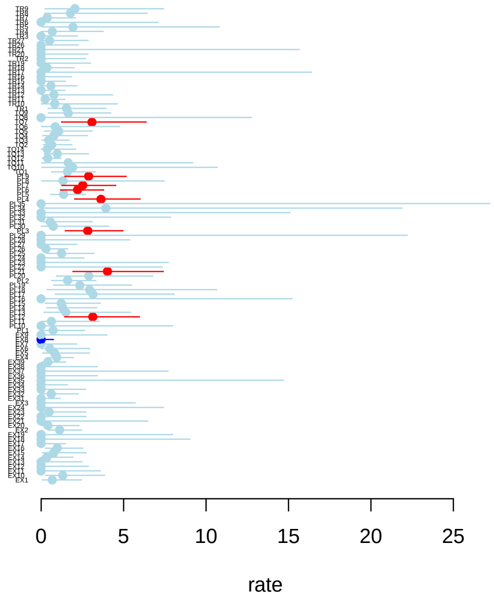

Figure 5: 95% Confidence interval for log relative risk parameter λ_{ij} for Nut allergies (four postcode sectors omitted due to very small population), those entirely above one highlighted in red, those entirely below one highlighted in dark blue

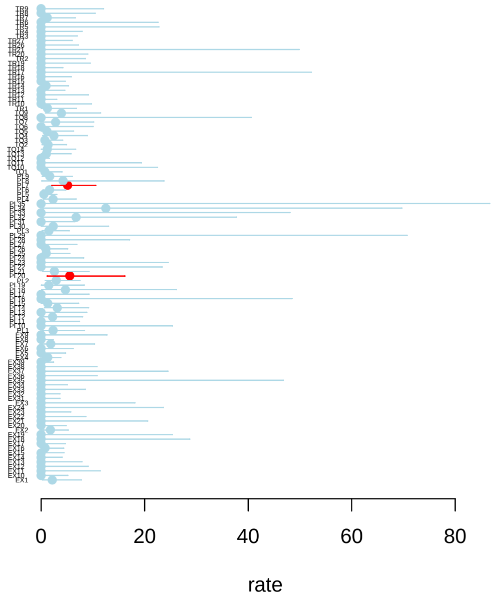

Figure 6: 95% Confidence interval for log relative risk parameter λ_{ij} for Fruit allergens (four postcode sectors omitted due to very small population), those entirely above one highlighted in red, those entirely below one highlighted in dark blue

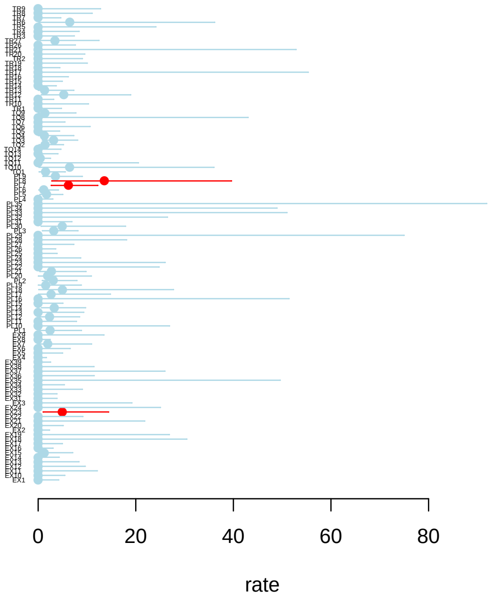

Figure 7: 95% Confidence interval for log relative risk parameter λ_{ij} for Latex allergies (four postcode sectors omitted due to very small population), those entirely above one highlighted in red, those entirely below one highlighted in dark blue

1.3 Checking the Poisson assumption

A number of supplementary R libraries intended for spatial analysis (Bivand, 2009; Gómez-Rubio, 2005) were used to complete the spatial analysis.

Central to any furthe investigation is a check as to whether the Poisson assumption in equation (1) is tenable.

1.3.1 Overdispersion- χ^2 test

First, we wish to determine whether the relative risks are homogenous i.e.,

$H_0 : \lambda_1 = \lambda_2 = \cdots = \lambda_n$

$H_A : \lambda_i$ cannot be considered homogenous

This can be examined by a simple χ^2 test:

$$\chi_j^2 = \sum_{i=1}^{n} \frac{(O_{ij} - r_j E_{ij})^2}{r_j E_{ij}}$$

1.3.2 Overdispersion: Potthoff and Whittinghill test

Secondly, we can perform a more focussed test proposed by Potthoff and Whittinghill (1966) that the relative risk parameters λ_{ji} are homogenous for a given allergy j against the alternative that the λ_{ij} are better considered as realisations from a Gamma distribution (in other words, that the data follow a Negative Binomial distribution rather than a Poisson distribution).

$H_0 : \lambda_{j1} = \lambda_{j2} = \cdots = \lambda_{jn}$

$H_A : \lambda_{ji} \sim Gamma(\lambda_j^2/\sigma_j^2, \lambda_j/\sigma_j^2)$

The test statistic is given by:

$$PW_j = \sum_{i=1}^{n} E_{ij} \sum_{i=1}^{n} \frac{O_{ij}(O_{ij} - 1)}{E_{ij}}$$

1.3.3 Overdispersion: spatial structuring

Finally, we consider the most likely reason for non-homogeneity in risks by postcode sector would be due to some kind of spatial structuring. We therefore use Moran's I statistic (as implemented by Cliff and Ord, 1981) as a test of this. If W_{ik} is a matrix whose entries equal 1 when area i is adjacent to area k, and 0 otherwise we have:

$$I_j = \frac{n \sum_{i=1}^{n} \sum_{k=1}^{n} W_{ik}(r_{ij} - \bar{r_j})(r_{kj} - \bar{r_j})}{(\sum_{i=1}^{n} \sum_{k=1}^{n} W_{ik}) \sum_l (r_{lj} - \bar{r_j})^2}$$

This is similar to a correlation coefficient and can be used as a measure of similarity between adjacent areas. As noted by Bivand *et al.* (2008), we apply the test to the SMR so that we don't detect spatial clustering caused by the population.

1.4 Results

We examine the three tests applied to each allergy condition in turn.

1.4.1 Airborne

There were $n = 623$ reported cases with Airborne allergies.

```
Chi-square test for overdispersion

        Type of boots.: parametric
        Model used when sampling: Multinomial
        Number of simulations: 999
        Statistic:  614.6782
        p-value :  0.001
Potthoff-Whittinghill's test of overdispersion

        Type of boots.: parametric
        Model used when sampling: Multinomial
        Number of simulations: 999
        Statistic:  708129.7
        p-value :  0.001
Moran's I test of spatial autocorrelation

        Type of boots.: parametric
        Model used when sampling: Negative Binomial
        Number of simulations: 99
        Statistic:  0.6225485
        p-value :  0.01
```

The results of the tests suggest that the data are indeed over-dispersed, and that there is spatial structuring within the data.

1.4.2 Pollen

There were $n = 318$ reported cases with Pollen allergies.

```
Chi-square test for overdispersion

        Type of boots.: parametric
        Model used when sampling: Multinomial
        Number of simulations: 999
        Statistic:  349.1539
        p-value :  0.001
Potthoff-Whittinghill's test of overdispersion

        Type of boots.: parametric
```

```
        Model used when sampling: Multinomial
        Number of simulations: 999
        Statistic:  178604.5
        p-value :  0.001
Moran's I test of spatial autocorrelation

        Type of boots.: parametric
        Model used when sampling: Negative Binomial
        Number of simulations: 99
        Statistic:  0.4288379
        p-value :  0.01
```

The results of the tests suggest that the data are indeed over-dispersed, and that there is spatial structuring within the data.

1.4.3 Food

There were $n = 410$ reported cases with Food allergies.

```
Chi-square test for overdispersion

        Type of boots.: parametric
        Model used when sampling: Multinomial
        Number of simulations: 999
        Statistic:  315.7686
        p-value :  0.001
Potthoff-Whittinghill's test of overdispersion

        Type of boots.: parametric
        Model used when sampling: Multinomial
        Number of simulations: 999
        Statistic:  260432.7
        p-value :  0.001
Moran's I test of spatial autocorrelation

        Type of boots.: parametric
        Model used when sampling: Negative Binomial
        Number of simulations: 99
        Statistic:  0.5520697
        p-value :  0.01
```

The results of the tests suggest that the data are indeed over-dispersed, and that there is spatial structuring within the data.

1.4.4 Seafood

There were $n = 71$ reported cases with Seafood allergies.

```
Chi-square test for overdispersion

        Type of boots.: parametric
        Model used when sampling: Multinomial
        Number of simulations: 999
        Statistic:  122.2056
        p-value :  0.194
Potthoff-Whittinghill's test of overdispersion

        Type of boots.: parametric
        Model used when sampling: Multinomial
        Number of simulations: 999
        Statistic:  6946.07
        p-value :  0.042
Moran's I test of spatial autocorrelation

        Type of boots.: parametric
        Model used when sampling: Negative Binomial
        Number of simulations: 99
        Statistic:  0.08713429
        p-value :  0.21
```

There is no evidence from the χ^2 test to indicate general overdispersion, although the Potthoff-Whittinghill test preferred H_A, that the Poisson rate paramters were Gamma distributed. There is also no significant evidence of spatial structuring. The number of cases is however rather small.

1.4.5 Nuts

There were $n = 223$ reported cases with Nut allergies.

```
Chi-square test for overdispersion

        Type of boots.: parametric
        Model used when sampling: Multinomial
        Number of simulations: 999
        Statistic:  221.2025
        p-value :  0.009
Potthoff-Whittinghill's test of overdispersion

        Type of boots.: parametric
        Model used when sampling: Multinomial
        Number of simulations: 999
        Statistic:  79969.73
        p-value :  0.001
Moran's I test of spatial autocorrelation
```

```
        Type of boots.: parametric
        Model used when sampling: Negative Binomial
        Number of simulations: 99
        Statistic:  0.4410051
        p-value :  0.01
```

The results of the tests suggest that the data are indeed over-dispersed, and that there is spatial structuring within the data.

1.4.6 Fruit

There were $n = 70$ reported cases with Fruit allergies.

```
Chi-square test for overdispersion

        Type of boots.: parametric
        Model used when sampling: Multinomial
        Number of simulations: 999
        Statistic:  121.3014
        p-value :  0.22
Potthoff-Whittinghill's test of overdispersion

        Type of boots.: parametric
        Model used when sampling: Multinomial
        Number of simulations: 999
        Statistic:  6851.06
        p-value :  0.032
Moran's I test of spatial autocorrelation

        Type of boots.: parametric
        Model used when sampling: Negative Binomial
        Number of simulations: 99
        Statistic:  0.1297910
        p-value :  0.09
```

There is no evidence from the χ^2 test to indicate general overdispersion, although the Potthoff-Whittinghill test preferred H_A, that the Poisson rate paramters were Gamma distributed. There is also no significant evidence of spatial structuring. The number of cases is however rather small.

1.4.7 Latex

There were $n = 66$ reported cases with Latex allergies.

```
Chi-square test for overdispersion

        Type of boots.: parametric
```

```
        Model used when sampling: Multinomial
        Number of simulations: 999
        Statistic:  188.842
        p-value :  0.026
Potthoff-Whittinghill's test of overdispersion

        Type of boots.: parametric
        Model used when sampling: Multinomial
        Number of simulations: 999
        Statistic:  10222.77
        p-value :  0.001
Moran's I test of spatial autocorrelation

        Type of boots.: parametric
        Model used when sampling: Negative Binomial
        Number of simulations: 99
        Statistic:  0.2951043
        p-value :  0.01
```

Interestingly, with the smallest sample size the results of these tests suggest that the data are indeed over-dispersed, and that there is spatial structuring within the data.

1.5 Spatial Smoothing

Marshall (1991) proposed an Empirical Bayes estimator assuming that the relative risks λ_{ij} have some common prior mean μ_j and prior variance σ_j^2. In order to avoid making distributional assumptions about λ_j results are given using moment estimators such that:

$$\lambda_{ij} = (1 - C_{ij})r_j + C_{ij}SMR_{ij}$$

where

$$C_ij = \frac{s^2 - r_j/\bar{E}_j}{s^2 - r_j/\bar{E}_j + r_j/E_ij}$$

We use a local smoothing, where r_j is estimated only for postcode sectors adjacent to postcode sector i in order to produce "smoothed" maps of the allergy risk. The idea is that where numbers are small, we average our uncertainty across neigbours who are thought to have a similar underlying risk.

2 References

Tomas Aragon, *et al.* (2010) *Applied Epidemiology Using R.* Available at http://www.epitools.net accessed 4/2/2010

Bailey T, Gatrell A (1995) *Interactive Spatial Data Analysis*, Harlow: Longman

Bivand R (2009) (with contributions by Luc Anselin, Renato AssunÃğÃčo, Olaf Berke, Andrew Bernat, Marilia Carvalho, Yongwan Chun, Bjarke Christensen, Carsten Dormann, StÃl'phane Dray, Rein Halbersma, Elias Krainski, Nicholas Lewin-Koh, Hongfei Li, Jielai Ma, Giovanni Millo, Werner Mueller, Hisaji Ono, Pedro Peres-Neto, Gianfranco Piras, Markus Reder, Michael Tiefelsdorf and and Danlin Yu). *spdep: Spatial dependence: weighting schemes, statistics and models.* R package version 0.4-56. http://CRAN.R-project.org/package=spdep

Cliff, A. D., Ord, J. K. (1981) *Spatial processes*, Pion

Diggle, P (1990) "A point process modelling approach to raised incidence of a rare phenomenon in the vicinity of a prespecified point" *Journal of the Royal Statistical Society A* **153**:359-362

Leslie Day (1992), "Simple SAS macros for the calculation of exact binomial and Poisson confidence limits." *Comput Biol Med*, **22**:351-361

V. Gómez-Rubio; J. Ferrándiz-Ferragud; A. López-Quílez (2005). "Detecting clusters of disease with R" *Journal of Geographical Systems* **7**:189-206

Kelsall, J.E. and Diggle, P.J. (1995a) "Kernel estimation of relative risk" *Bernoulli* **1**:3-16

Kelsall, J.E. and Diggle, P.J. (1995b) "Non-parametric estimation of spatial variation in relative risk" *Statistics and Medicine* **14**:559-573

Marshall R M (1991) "Mapping disease and mortality rates using Empirical Bayes Estimators", *Applied Statistics*, **40**: 283-294;

Moran, P.A.P (1948) "The interpretation of statistical maps", *Journal of the Royal Statistical Society Series B*: **10**:243-251

R Development Core Team (2009) *R: A language and environment for statistical computing. R Foundation for Statistical Computing*, Vienna, Austria. ISBN 3-900051-07-0, URL http://www.R-project.org.

Rowlingson B., Diggle,P., adapted, packaged for R by Roger Bivand, pcp functions by Giovanni Petris and goodness of fit by Stephen Eglen (2009). *splancs: Spatial and Space-Time Point Pattern Analysis. R package version 2.01-25.* http://CRAN.R-project.org/package=splancs

Potthoff, R. F. and Whittinghill, M.(1966) "Testing for Homogeneity: The Poisson Distribution" *Biometrika* **53**, 183-190
